# Characterization of mycobacteria isolated from the Brazilian Atlantic Forest: a public health and bioprospection perspective

**DOI:** 10.3389/fmicb.2025.1558006

**Published:** 2025-04-25

**Authors:** Leandro Santiago Emmerick, Marcos Gustavo Araujo Schwarz, Paloma Rezende Corrêa, Sindy Licette Piñero, Leonardo Henrique Ferreira Gomes, Ana Maria Mazotto Almeida, Richard Hemmi Valente, Wim Maurits Sylvain Degrave, Leila Mendonça-Lima

**Affiliations:** ^1^Laboratório de Genômica Aplicada e Bioinovações, Instituto Oswaldo Cruz, Fundação Oswaldo Cruz, Rio de Janeiro, Brazil; ^2^Laboratório de Biologia Molecular Aplicada à Micobactérias, Instituto Oswaldo Cruz, Fundação Oswaldo Cruz, Rio de Janeiro, Brazil; ^3^Laboratório de Alta Complexidade, Unidade de Pesquisa Clínica, Instituto Fernandes Figueira, Fundação Oswaldo Cruz, Rio de Janeiro, Brazil; ^4^Laboratório de Biocatálise Microbiana, Instituto de Microbiologia Paulo de Góes, Federal University of Rio de Janeiro, Cidade Universitária, Rio de Janeiro, Brazil; ^5^Laboratório de Toxinologia, Instituto Oswaldo Cruz, Fundação Oswaldo Cruz, Rio de Janeiro, Brazil

**Keywords:** nontuberculous mycobacteria, Brazilian Atlantic Forest, cellulase, protease, mycobacterium shared antigens

## Abstract

The Mycobacterium genus remains highly relevant today due to the rising incidence of tuberculosis and opportunistic infections caused by environmental mycobacteria. While much is known about *M. tuberculosis*, *M. leprae* and *M. bovis*, studies focusing on environmental mycobacteria remain limited. These microorganisms are globally distributed and have been identified in diverse biomes, including the Atlantic Forest. This study aims to provide a characterization of four mycobacterial strains isolated from the Atlantic Forest, assessing their metabolic capabilities and biotechnological potential. We investigated the presence of cellulases and proteases and conducted an initial profiling of secreted proteins. Furthermore, the examination of shared antigens and infection kinetics within macrophages offered insights into the ecological and pathogenic potential of these isolates. From a public health perspective, antigenic similarities between these environmental microorganisms and the BCG vaccine strain may influence the efficacy of BCG in protecting against diseases such as tuberculosis. Continued research on these and other environmental isolates, particularly within Brazil’s highly biodiverse ecosystems, holds promise for advancing scientific knowledge and contributing to human health.

## 1 Introduction

The Brazilian biomes harbor an exceptional biological diversity, characterized by a high species richness and remarkable endemism. Notably, the Atlantic Forest stands out as one of the world’s 25 biodiversity hotspots (Myers et al., 2000). Conservation efforts within this biome, including establishing Conservation Units like the Serra dos Órgãos National Park (PARNASO), the country’s third oldest park, have been instrumental in preserving its ecological integrity. Scientific endeavors within these protected areas have led to the establishment of an Atlantic Forest Bacteria Collection at the Oswaldo Cruz Institute (CBMA/IOC) (Bruce et al., 2010).

Mycobacteria, prominent for species such as *Mycobacterium tuberculosis* and *M. leprae*, causative agents of tuberculosis and leprosy, respectively, comprise a significant microbial group (World Health Organization, 2023). Beyond these pathogenic strains, non-tuberculous mycobacteria (NTM) exhibit a free-living lifestyle and sporadically pose infection risks to humans and other organisms (Conyers and Saunders, 2024). With the rising incidence of NTM infections, investigations have intensified, focusing on taxonomic elucidation and biological characterization (Zhang et al., 2024).

The remarkable adaptability of mycobacteria to diverse habitats stems from several key factors. These include the hydrophobic nature of their mycolic acid-rich cell envelope, endowing resistance to antibiotics, surface adherence, and biofilm formation (Meyer and Bramkamp, 2024; Patel et al., 2024). Their slow growth dynamics, driven by low protein synthesis rates and standard metabolic activities, also facilitate acclimatization to fluctuating environmental conditions. Furthermore, mycobacteria demonstrate resilience to various stressors, such as pH alterations, nutrient availability, temperature fluctuations, and oxygen levels, enabling survival across a spectrum of anthropogenic and natural environments (Honda, 2023; khan et al., 2023; Dechow and Abramovitch, 2024).

Traditional molecular identification methods, employing genetic markers (16S rRNA, *hsp65*, *sodA*, and *recA*) and intergenic regions (e.g., ITS), have encountered limitations due to mycobacterial species’ clonal nature and genome conservation. Advancements in next-generation sequencing have revolutionized species identification and taxonomic classification by enabling comprehensive genome analysis (Zhang et al., 2024). Several research groups have been working on genomic sequencing and data analysis from NTM recovered from patients (Machado et al., 2021; Cudahy et al., 2024), often clinically mistaken for other mycobacterial infections such as tuberculosis, and those isolated from environmental samples (Morgado and Vicente, 2020, 2021). Many of these studies focus on identifying markers that facilitate rapid identification and aid in selecting therapeutic protocols while also seeking insights into the dynamics that enable these mycobacteria to cause diseases, considering both patient and bacterial perspectives (Machado et al., 2024).

Environmental mycobacteria from PARNASO, deposited at CBMA/IOC, have already been the subject of genomic studies, revealing a highly diverse mobilome. These studies also identified a novel integrative conjugative element and plasmids carrying the type VII secretion system (T7SS) (Morgado and Vicente, 2020). Although this study did not find evidence of antibiotic resistance or virulence genes within these mobile elements, there were indications of sequences from non-mycobacterial species, suggesting horizontal gene transfer (HGT) among species in the environment. This contrasts with what is known about this event in pathogenic specimens of the genus, where it is believed that HGT was more frequent in the group’s evolutionary past but has become rare in contemporary times, remaining inconclusive whether such an event still occurs (Xia, 2023). Therefore, it is hypothesized that these NTM possess an enhanced ability to acquire genetic material from the environment, thereby increasing their capacity to adapt to environmental changes, such as the transition to a human host.

This study entails the phenotypic characterization of four fast-growing mycobacterial isolates from Atlantic Forest soil in Rio de Janeiro. Investigations encompassed, among other assays, the examination of shared antigens and infection kinetics within macrophages, providing insights into these microorganisms’ ecological and pathogenic potential. Additionally, we conducted preliminary bioprospection assays to determine whether these microorganisms actively produce and secrete proteases and cellulases, since these enzymes have been identified in secreted form in other mycobacterial species, where they play roles in microbial physiology. Cellulase, for instance, is known to participate in the dynamics of cellulose-based biofilm formation in *M. tuberculosis*, thereby influencing virulence and other infection-related parameters (Chakraborty et al., 2021). For NTM, this enzyme class is believed to be involved in carbon source acquisition (Mon et al., 2024). Furthermore, these enzymes are of significant biotechnological interest, being currently employed in various industrial processes including food processing (Song et al., 2023) and bioethanol production (Barbosa et al., 2020).

## 2 Materials and methods

### 2.1 Microbial isolates and culture conditions

Isolates 226, 271, 293, and 294 were sourced from the Atlantic Forest Bacteria Collection (CBMA/IOC) and preserved in liquid nitrogen. Seed lots were prepared, quantified in colony-forming units (CFU), and used in the experimental procedures. The isolates were cultured in LB/Tw [Luria-Bertani medium supplemented with 0.05% (v/v) Tween 80], maintained under agitation at 22°C. Growth kinetics was monitored through optical density readings at 600 nm (OD_600nm_). Ziehl Neelsen staining was done using established protocols (Van Deun et al., 2008).

### 2.2 Biochemical tests

All biochemical tests were conducted using stationary-phase cultures, incubated at room temperature, with results observed after 14 days, except for *M. smegmatis* mc^2^155, which was cultured at 37°C and observed after 7 days, while nitrate reduction was observed after 14 days. Nitrate reduction: Isolates were cultured in test tubes containing a specific medium, inoculated with 200 μL, and results were observed after the addition of 400 μL of sulfanilic acid and 400 μL of *α*-naphthylamine. Citrate: Isolates were cultured in test tubes containing Christensen’s medium and observation was based on changes in medium coloration. Methyl Red (MR): Isolates were cultured in test tubes containing glucose broth medium, and the pH value was determined by adding 0.5 mL of methyl red pH indicator. Voges-Proskauer (VP): Isolates were cultured in test tubes containing glucose broth medium with an inoculum of 200 μL and observed after the addition of 1 mL of 40% NaOH and 0.5 mg of creatine, followed by agitation and incubation for 15 min at room temperature. Anaerobiosis: Isolates were cultured in test tubes containing a solid medium using the puncture technique and observed for growth under anaerobic conditions. Gelatin hydrolysis: Isolates were cultured in test tubes containing nutrient medium with 12% bacteriological gelatin (Difco), inoculated with 200 μL, and observed for 4 h at 4°C. NaCl tolerance: Isolates were cultured in test tubes containing nutrient medium with NaCl concentrations of 5% (w/v), 7% (w/v), or 10% (w/v), inoculated with 200 μL, and observed for growth in the nutrient medium for up to 5 days.

### 2.3 Electrophoresis and western blot analysis

Cells were harvested via centrifugation, resuspended in 1 mL of lysis buffer [50 mM HEPES/KOH pH 7.5, 10 mM MgCl_2_, 60 mM NH_4_Cl, and 10% (v/v) glycerol], and subjected to three lysis cycles (each lasting 1 min) using a Bead Beater apparatus (Bio101) with glass beads. The resultant clarified lysate (total protein fraction) was obtained following centrifugation at 15,000 *g* for 10 min. The culture filtrate underwent precipitation with trichloroacetic acid (TCA) followed by resuspension in a solution containing 8 M urea and 2% (w/v) CHAPS for the fraction of secreted proteins. Total proteins were quantified using NanoDrop.

Twenty μg of protein were resolved via 15% SDS-PAGE prior to western blotting and immunodetection based on polyclonal antibodies against several mycobacterial proteins [Mpt64, Mpt70, Mpt83, GlnA1, HspX, Ag85, and cellulase (CelA1)]. In brief, proteins were transferred onto a nitrocellulose membrane (Hybond C, GE) using a Mini Trans-blot apparatus (Bio-Rad) at a constant voltage of 100 V for 1 h. The efficiency of protein transfer was assessed by reversible staining with MemCode (Pierce). Subsequently, the membrane was blocked overnight at 4°C in TBS-T [Tris-buffered saline supplemented with 0.05% (v/v) Tween 20] containing 10% (w/v) skim milk. Following washing with TBS-T, the membrane was incubated with mouse or rabbit (for Ag85) polyclonal antibody (1:1,000 for all, except for Ag85, which was 1:10,000) for 2 h, followed by incubation with HRP-conjugated goat anti-mouse (for all, except for Ag85) or anti-rabbit IgG (for Ag85) (1:10,000; Thermo Fisher Scientific), for 1 h. After each antibody incubation, the membrane was washed three times with TBS-T, followed by three washes with TBS. All antibody incubations were conducted in a 5% (w/v) skim milk solution in TBS-T. The blots were developed using the SuperSignal kit (Pierce), following the manufacturer’s protocol.

### 2.4 Protease and cellulase zymography

The production of proteases and cellulases was assessed in the total lysate using polyacrylamide gel zymography. For the protease assay, 0.1% (w/v) porcine gelatin (Sigma) was used as the substrate. For the cellulase assay, 0.2% (w/v) CMC (carboxymethyl cellulose) was used as the substrate. Twenty micrograms of mixed proteins in native sample buffer were applied and subjected to electrophoresis on 12% SDS-PAGE gels in Laemmli running buffer at 200 V. After electrophoresis, the gel was washed twice for 15 min in 2.5% Triton X-100 solution, followed by 15 min in Milli-Q water, and then incubated for 30 min in 50 mM potassium acetate buffer at 28°C. Finally, the gelatin gel was stained with Coomassie Brilliant Blue-R250 solution and destained with a destaining solution. The gel for cellulase detection was stained with a 0.1% Congo red solution and destained with 1 M NaCl.

### 2.5 Macrophage infection assay

THP-1 derived macrophages were obtained as previously described (Corrêa et al., 2023) and infected with the isolates using a MOI (multiplicity of infection) of 10:1. After 4 h, the culture medium was removed, and the eukaryotic cells were washed three times with RPMI medium, followed by the addition of preheated RPMI supplemented with 10% fetal bovine serum (FBS). The medium exchange was performed every 24 h. The kinetics of bacterial intracellular viability was evaluated at 4, 24, 48, and 72 h post-infection. At the selected time points, infected macrophages were lysed with 0.05% SDS, and the bacteria were collected and centrifuged for 10 min at 16,000 *g*. The supernatant was discarded, and the bacterial pellet was resuspended in 1 mL of LB/Tw medium. Serial dilutions were made from this bacterial suspension and plated for CFU counting.

### 2.6 Secretome proteomics

The isolates were grown in LB/Tw medium for 48 h at 22°C. The bacteria were discarded after centrifugation at 5,000 *g* for 15 min, and the supernatant was filtered through a 0.22 μm PVDF membrane (Millipore). The proteins from the culture filtrate were precipitated by adding 85% (w/v) ammonium sulfate and centrifuged at 5,000 *g* for 15 min. The supernatant was discarded, and the precipitate was resuspended in 10 mM Tris–HCl buffer, pH 7.2. The material was dialyzed three times against the same resuspension buffer.

For shotgun proteomics analysis, 50 μg of protein from each isolate was submitted to trypsin digestion as described previously (Almeida et al., 2020). Peptide samples were desalted using ZipTip C18 following the manufacturer’s instructions (Merck Millipore, USA) and resuspended in 10 μL of 1% formic acid. Each isolate’s secretome was analyzed in triplicate by nanoLC-MS/MS. For that effect, nanochromatography was performed on an EASY-nLC II instrument (Thermo Scientific, USA) as described elsewhere (Nicolau et al., 2017), with the following modifications: only one microliter was injected per technical replicate, the self-packed column was shorter (10 cm), and a faster gradient (2 to 40% B during 52 min) was used. Eluted peptides were directly introduced into a nanoESI Q Exactive Plus mass spectrometer (Thermo, USA) for high-resolution data acquisition (Almeida et al., 2023).

All MS/MS spectra were analyzed using PEAKS Studio 11.5 build 20231206 (Bioinformatics Solutions, Canada). After data refinement (mass correction and chimera spectra association), PEAKS *DE NOVO* analysis was run assuming trypsin digestion, with a fragment ion mass tolerance of 0.02 Da and a parent ion tolerance of 10 ppm. Cysteine carbamidomethylation was set as a fixed modification, and the following variable modifications were searched: deamidation at N/Q and carbamidomethylation at D/H/K/E and the N-terminus; a maximum of 3 variable modifications per peptide was allowed. PEAKS DB analysis was performed using these same parameters plus the possibility of up to two missed enzyme cleavages and semi-specific cleavage of the peptides. Searches were made against the “MycobacteriaAtlanticForest&Ecoli_20240723_Noredundancy” database (described below) and a universal protein contaminant database (Frankenfield et al., 2022); the deep learning boost option was enabled. False discovery rates (FDR) equal to or less than 1% were established at peptide-spectrum match, peptide, and protein levels; only protein identifications with at least 2 unique peptides were taken into consideration. The database used in this work was assembled as follows. The GenBank and RefSeq protein sequence databases for strains CBMA 226 (taxid 2606611), CBMA 271 (taxid 2606608), CBMA 293 (taxid 2606602), and CBMA 294 (taxid 2606604) were downloaded from https://www.ncbi.nlm.nih.gov/datasets/genome/. All protein entries from these eight databases displaying 100% sequence identity and that were a subset of a longer sequence were merged into a single entry using the Generate Search DB option from the PatternLab for Proteomics V (version 5.0.0.187) public domain software (Santos et al., 2022), generating a non-redundant database for these mycobacteria with 24,162 protein entries. Furthermore, to improve our false positive identification detection, we added the non-redundant version (5,554 entries) of an *Escherichia coli* database (UniProt Proteome ID UP000279837). The final database, named “MycobacteriaAtlanticForest&Ecoli_20240723_Noredundancy,” contained 29,716 entries.

## 3 Results

### 3.1 Growth in axenic media and overall morphology

**Figure 1:**
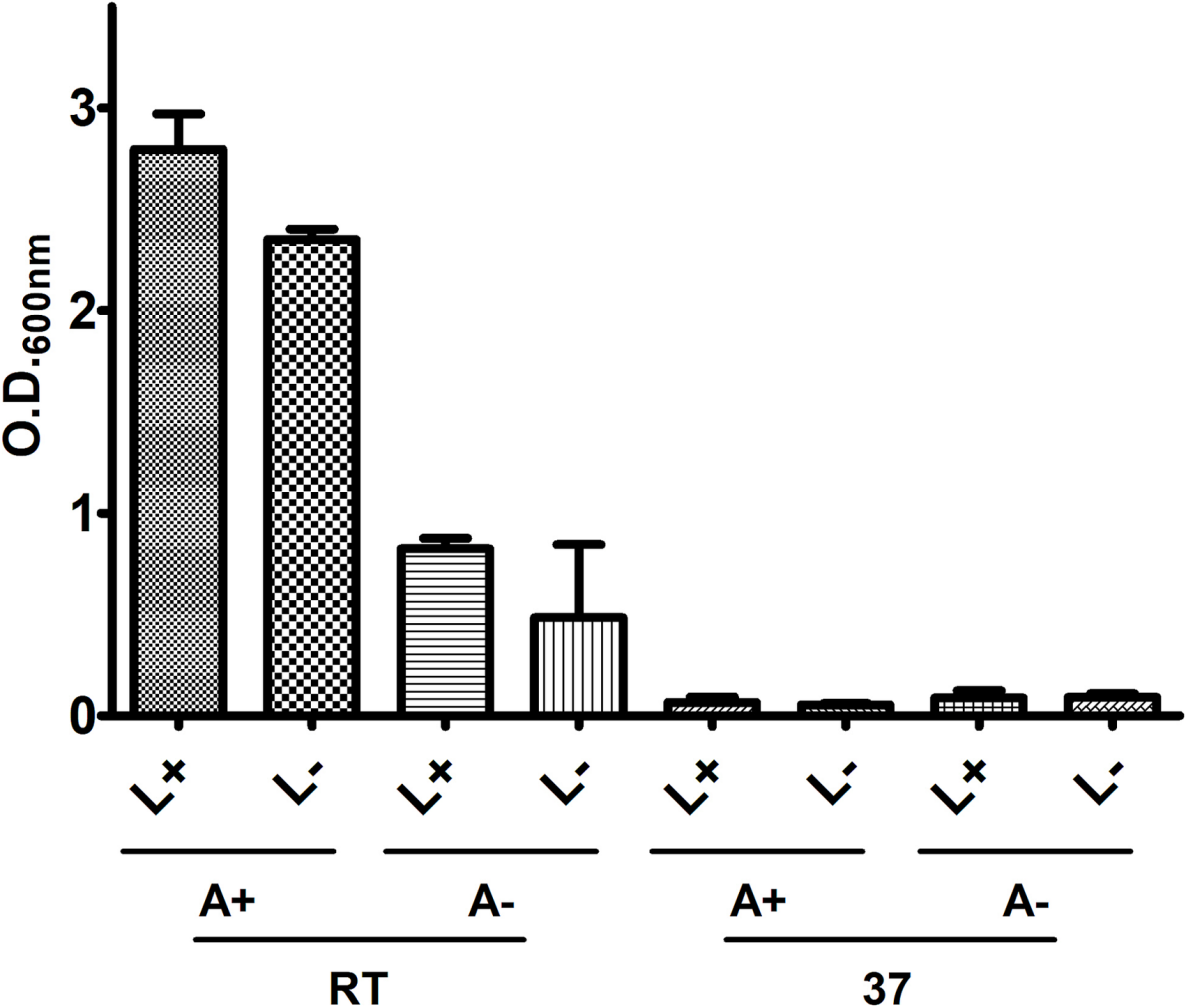
Assessment of light, agitation, and temperature on the *in vitro* growth of 226. Bacteria were cultivated in LB/Tw, and growth was measured by O.D._600nm_ under different conditions of temperature (room temperature – RT, or at 37°C), agitation (with – A+, or without – A-), and light presence (with – L+, or without L-).

To standardize the cultivation methodology of CBMA isolates, strain 226 was employed to assess various incubation parameters, including temperature [room temperature (22°C) or 37°C], agitation (200 rpm), and light exposure (Figure 1). Following a 7-day cultivation period in LB/Tw, it was observed that growth occurred at ambient temperature but not at 37°C. Agitation was found to promote growth and reduce the likelihood of clumping, whereas light exposure did not significantly affect this variable. Consequently, growth standardization in this culture medium was established at ambient temperature under agitation.

To assess the growth kinetics, bacterial growth curves were conducted in LB/Tw culture medium, monitoring O.D._600nm_. The culture integrity was periodically verified through Ziehl-Neelsen staining, followed by observation under a light microscope.

**Figure 2:**
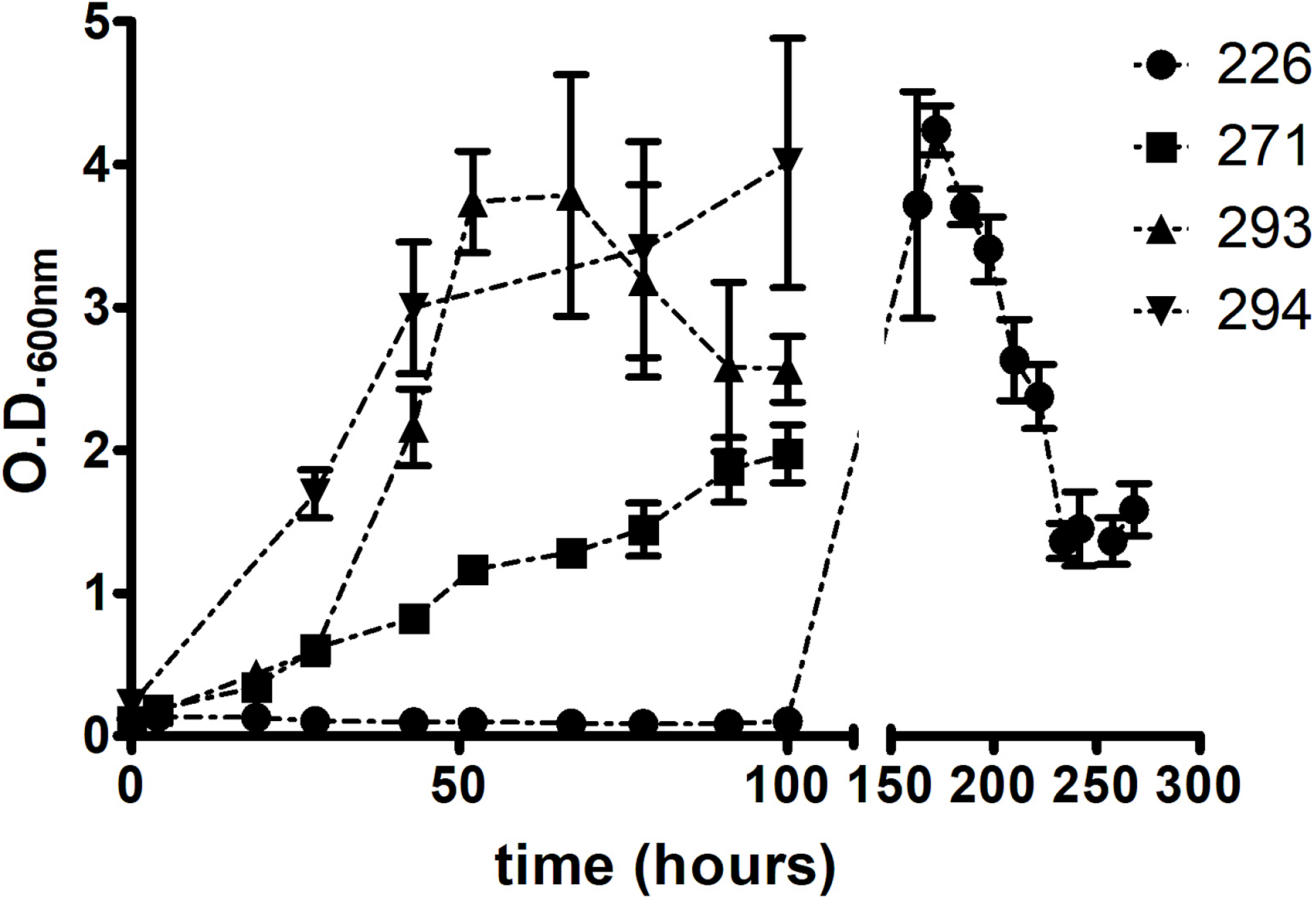
Axenic growth patterns of the four isolates on LB/Tw medium.

Isolate CBMA 226 exhibited an atypical behavior compared to the others (Figure 2). During the initial 100 h, there was a slight decrease in O.D._600nm_, followed by growth after approximately 150 h, reaching maximum O.D._600nm_ around 170 h of growth. Subsequently, it decreased over the next 70 h and stabilized in the last 30 h evaluated. No clumping was observed during the growth period. Isolates CBMA 293 and 294 displayed very similar bacterial growth patterns, whereas CBMA 271 displayed a slower growth rate.

**Figure 3:**
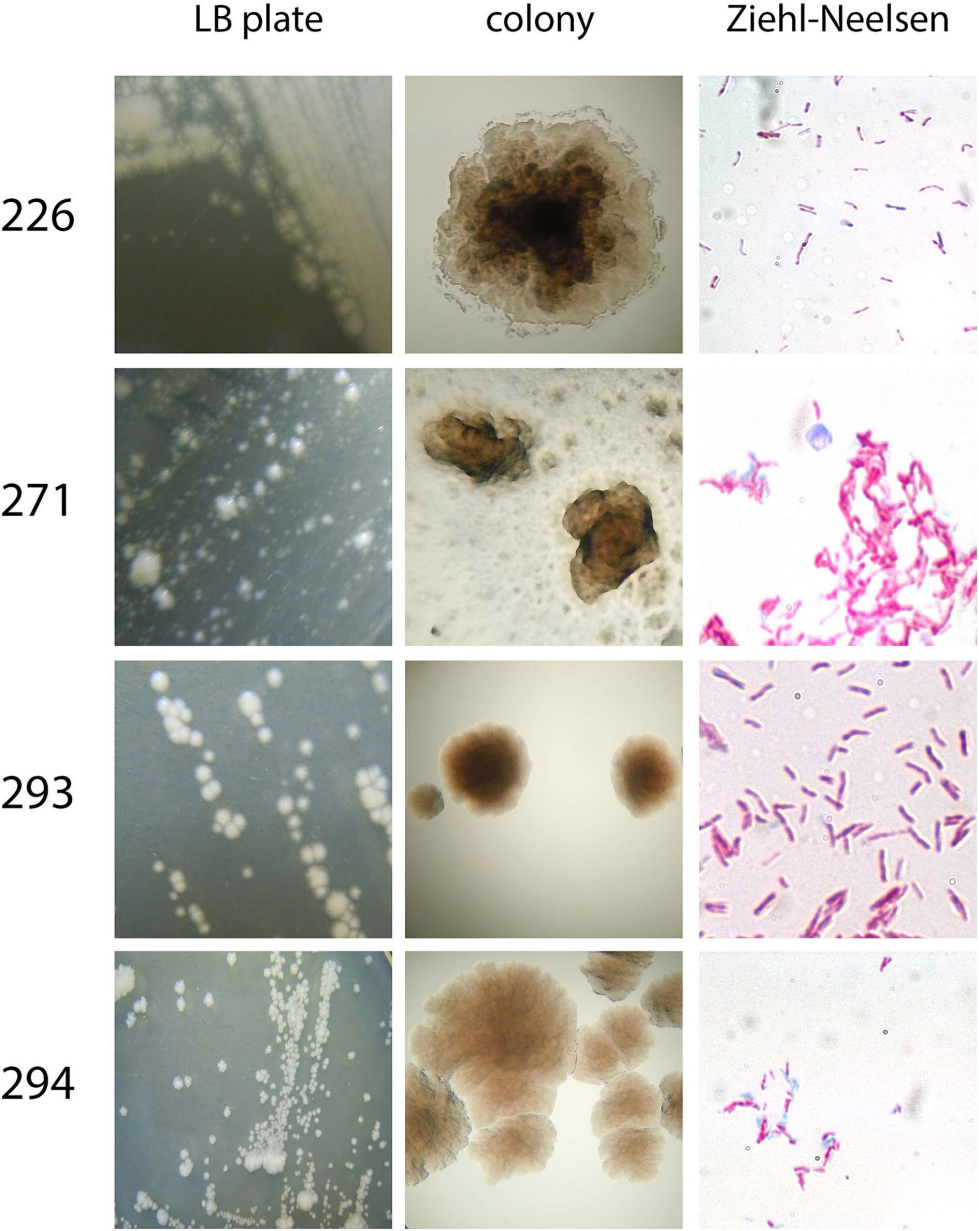
Overall morphology of the four isolates. Colony morphology was assessed after plating on LB/agar in a macroscopic (LB plate and colony) manner. Aside from that, Ziehl-Neelsen staining was performed to assess bacteria morphology, clumping tendency, and acid-alcohol-resistance.

To examine colony morphology, isolates were plated from the original culture provided by CBMA/IOC onto LB agar plates and incubated at room temperature, shielded from light. Microscopic examination of the isolates’ morphology was conducted using a light microscope following staining with the Ziehl-Neelsen technique. It was observed that all isolates exhibited a tendency to form clumps, and they all exhibited a pink coloration under microscopy (Figure 3).

General aspects of bacterial metabolism among the isolates were assessed via various biochemical tests commonly utilized for bacterial species identification and characterization, in comparison with *M. smegmatis* mc^2^155 (Table 1). It was observed that all tested mycobacteria, with the exception of isolate 271, demonstrated the ability to reduce nitrate, indicating their ability to utilize this compound as a terminal electron acceptor. Moreover, only isolate 293 exhibited the production of proteolytic enzymes capable of gelatin hydrolysis. Additionally, while *M. smegmatis* could thrive in the presence of up to 5% (w/v) NaCl, isolates 226, 271, and 293 showed intolerance to this saline concentration. Conversely, isolate 294 displayed halotolerance, as it could grow in up to 10% (w/v) NaCl.

**Table 1:** Biochemical tests.

Biochemical test	226	271	293	294	mc2
Nitrate	+	−	+	+	+
Citrate	+	+	+	+	+
Methyl red	−	−	−	−	−
Voges-Proskauer	−	−	−	−	−
Anaerobiosis	−	−	−	−	−
Gelatin	−	−	+	−	−
5% NaCl	−	−	−	+	+
7% NaCl	−	−	−	+	−
10% NaCl	−	−	−	+	−

CBMA/IOC isolates were incubated at room temperature and results were revealed after 14 days for all tests. *M. smegmatis* mc2 was used for comparative purposes, and it was incubated at 37°C, and results were revealed after 7 days, except for the nitrate test (14 days). (+) positive, (−) negative.

### 3.2 BCG shared antigens

**Figure 4:**
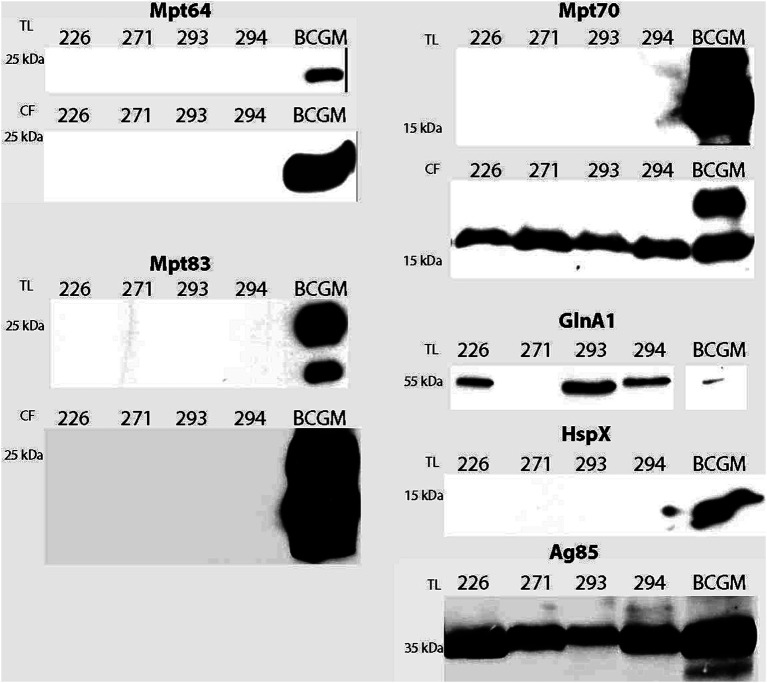
Detection of shared antigens with BCG. Prior to the western blot, 20 μg of proteins from each sample were resolved on 12% SDS-PAGE. Analysis was performed with total lysate (TL) and/or culture filtrate (CF) protein fractions from all isolates and *M. bovis* BCG Moreau (positive control). Polyclonal sera were previously produced in mice or rabbit against BCG Moreau homologs (Mpt64, Mpt70, Mpt83, GlnA1, HspX, Ag85).

To investigate the presence of antigens shared with species from the *M. tuberculosis* complex, western blot analysis with immunodetection of selected antigens was conducted on total lysates and/or culture filtrates of the studied isolates. Mpt64 and Mpt83 were not detected in either the total lysate or the culture filtrate (Figure 4). For all isolates, Mpt70 was detected in the culture filtrate but not in the total lysate. Interestingly, *M. bovis* BCG Moreau exhibited two bands near 20 kDa, whereas the isolates displayed only one band corresponding to Mpt70. For GlnA1, HspX, and Ag85, only total lysates were evaluated. Mpt83 and HspX were undetected in the isolates, while Ag85 was consistently detected in all isolates; GlnA1 was present in all isolates except for 271.

### 3.3 Protease and cellulase production

**Figure 5:**
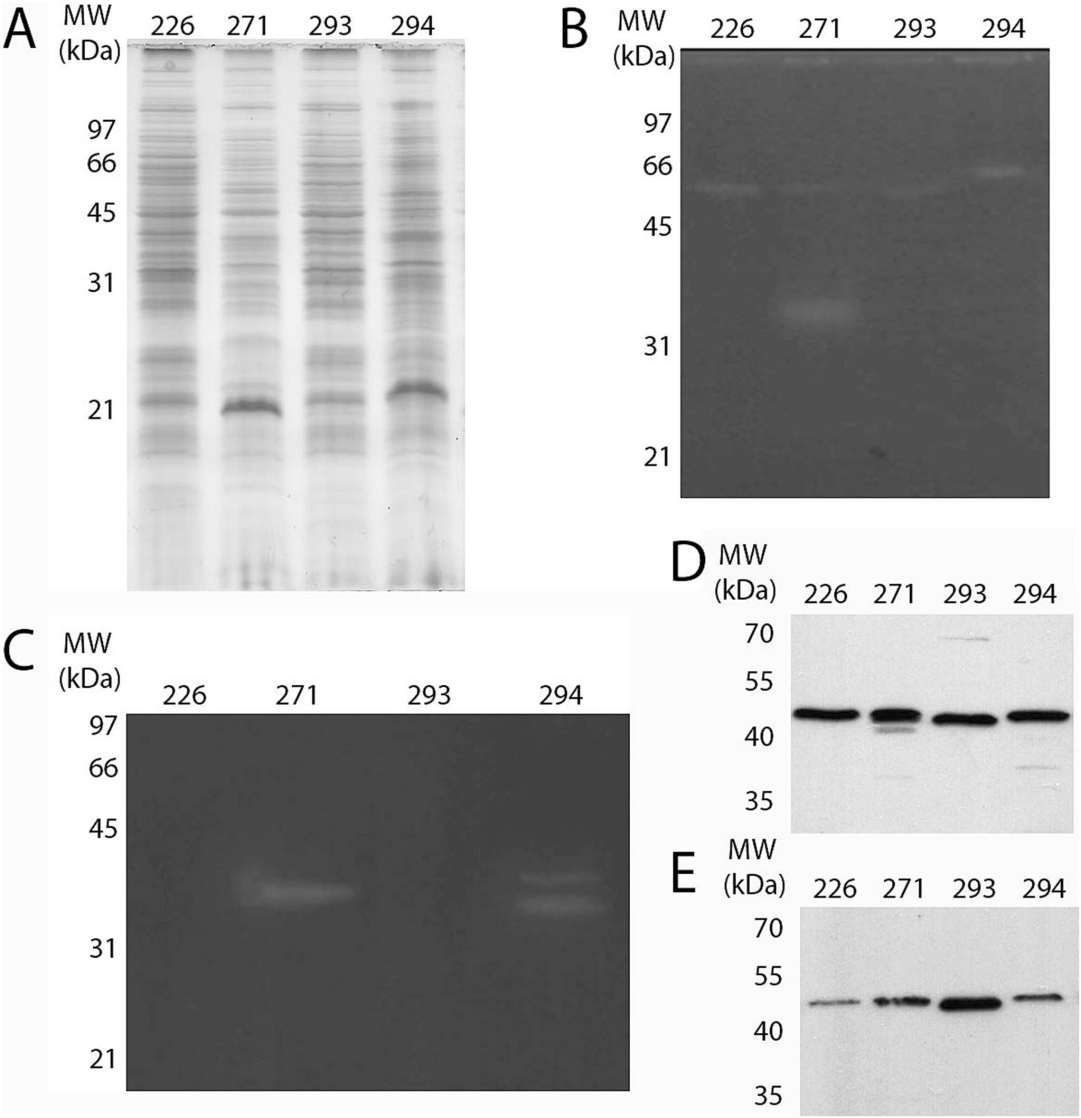
Detection of protease and cellulase in the protein extracts of the isolates. **(A)** Protein complexity from intracellular fraction of all four isolates was analyzed by SDS-PAGE 12% after staining with CBB R-250. Protease **(B)** and cellulase **(C)** activity was assessed by zymography in the intracellular protein fraction with respective substrates (gelatin and carboxymethylcellulose, respectively). Following SDS-PAGE 12%, excess SDS was removed and enzyme activity was visualized as described in the Methods section. Cellulase was also detected through western blot assay using α-CelA1 (1:1,000) as the primary antibody, both in **(D)** the intracellular and **(E)** the culture filtrate fractions.

The intracellular protein fraction of the isolates was examined to detect proteases and cellulases using gel zymography assays and western blot analysis with a mouse anti-CelA1 polyclonal antibody, specifically for cellulase detection. Both the intracellular protein fraction and the TCA-precipitated culture filtrate were utilized in the western blotting procedure. Results revealed that all assessed isolates exhibited minor proteolytic activity around 66 kDa, with isolate 271 also displaying slightly more pronounced activity near 35 kDa. Regarding cellulase detection, a band corresponding to cellulase activity was detected in 271 (a single band) and 294 (two bands), but all isolates had reacted bands with the anti-CelA1 antibody (Figure 5).

### 3.4 Macrophage infection

To evaluate the intracellular survival of these environmental isolates, a measure of their potential pathogenicity, a THP-1 human monocytic cell line was utilized as an infection model after differentiation into macrophages. *M. smegmatis* mc^2^155 served as a reference strain for comparative analysis.

**Figure 6:**
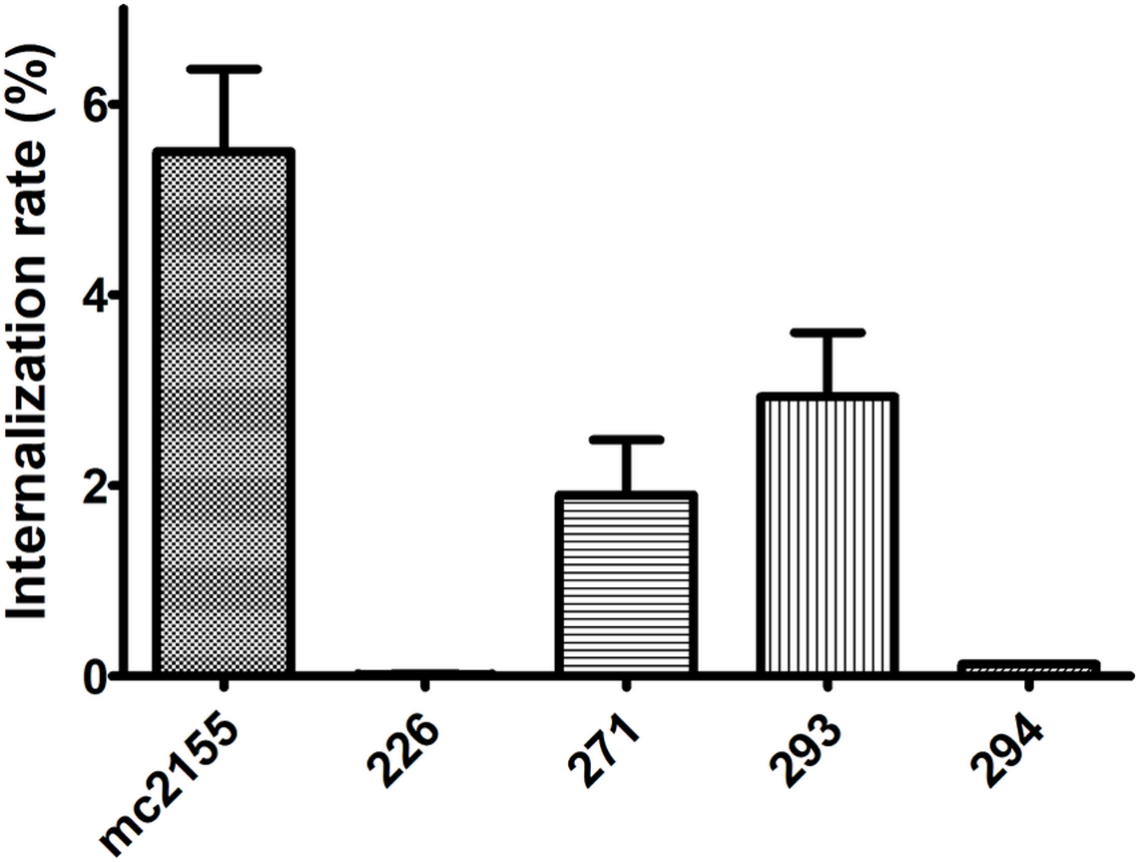
Internalization rate in a macrophage model of infection. All four isolates were able to infect THP-1 macrophages. Internalization rate is expressed as the number of bacteria associated with THP-1 cells at 4 h p.i. (post-infection)/number of bacteria in the initial inoculum.

The internalization rate of all isolates, relative to the initial bacterial quantity used for infection (2 × 10^6^ bacteria, MOI 10:1), was notably low, ranging from 5.5 to 1.5% for mc^2^155, 293, and 271, and less than 0.2% for 226 and 294 (Figure 6).

**Figure 7:**
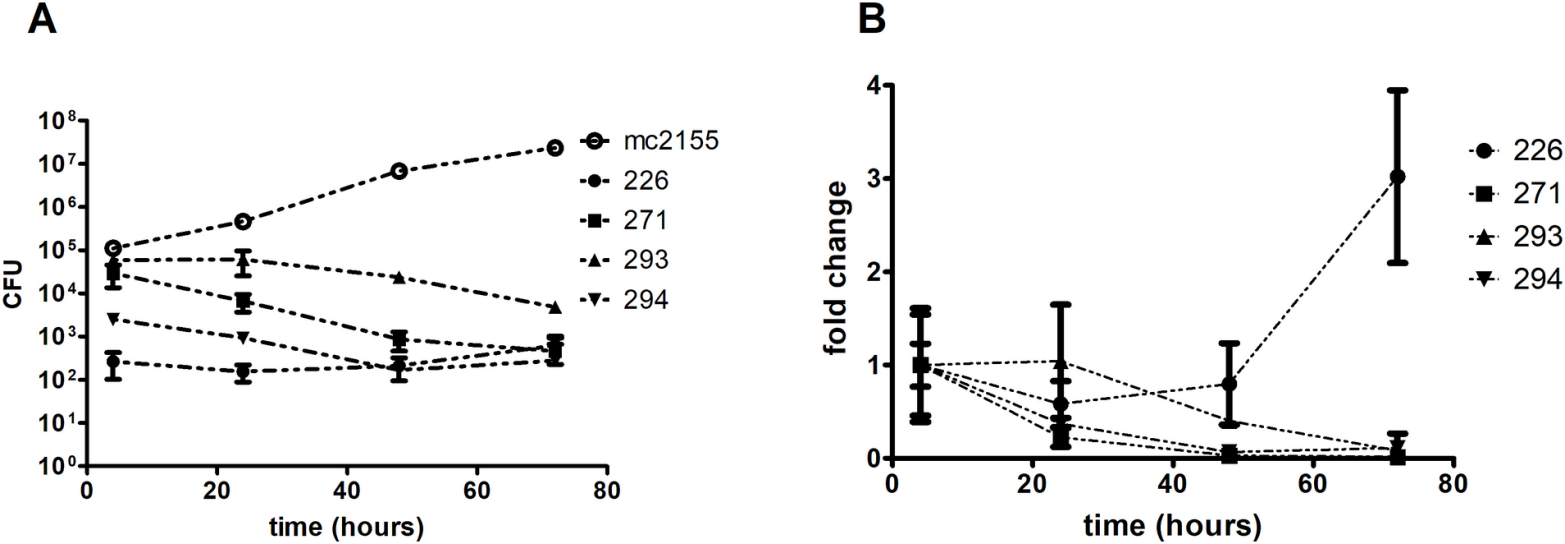
Isolates growth comparison in THP-1 derived macrophages. Macrophages were infected using a MOI of 10:1 for 4 h. After that, intracellular bacteria were collected through differential lysis and plated for CFU counting. *M. smegmatis* mc2 was used as a saprophyte control. **(A)** Growth kinetics show an overall tendency of growth decline for all environmental isolates, except for 226, emphasized when analyzing the **(B)** growth rate (fold change) compared to the initial bacterial input.

The viability of the environmental isolates and mc^2^155 was determined through CFU counting on LB agar at 4, 24, 48, and 72 h post-infection (Figure 7). The bacterial viability profile remained consistent over time for isolate 226. Isolates 293 and 294 showed an approximate 10-fold decline in bacterial count, whereas 271 exhibited a reduction of around 100-fold. In contrast, mc^2^155 displayed an average 10-fold increase in growth every 24 h (Figure 7A). A comparison of isolate growth rates with the initial bacterial quantity (at 4 h post-infection) revealed that isolate 226 exhibited an increase in later stages of infection, distinguishing it from the other isolates (Figure 7B).

### 3.5 Secretome analysis

The secretome analysis of the four isolates was conducted using a shotgun approach on a Q Exactive Plus instrument. We conducted a dual approach for protein identification: first, a database search using the MS/MS spectra against predicted proteins derived from the deposited genomes, and second, a Blast search using *de novo* sequenced peptides that did not find matches in the initial method, searching against the non-redundant protein sequences (nr) database, within the *Mycobacterium* sp. (taxid 1785) (DB.proteins and denovoOnly Tables, respectively, in the Supplementary material). Using database search parameters, proteins in the secretome of isolate 271 could not be identified. Therefore, identification for this sample was only achievable through Blast search using *de novo* sequenced peptides. Preliminary results identified some shared antigens with BCG, which were investigated by western blot assays. Mpt70 and GlnA1 were found in isolates 226 and 293. Noteworthy, the antigen 85 complex proteins were present in the secretome from 226, 293, and 294. Aside from these previously investigated antigens, others were also identified, like low molecular weight antigen MTB12, immunogenic protein MPT63, and 27 kDa antigen Cfp30.

In addition to secreted antigens, proteins with proteolytic activity (serine and metalloendopeptidases), cellulolytic enzymes, and others potentially valuable for biotechnological applications such as amidohydrolases, esterases, lipases, cutinases, peroxidases, and peptidases were identified (Tables in the Supplementary material; summarized in Table 2). Furthermore, homologs to proteins known for their importance in pathogenic species as virulence factors were identified in the isolates, like 6 kDa early secretory antigenic target (ESAT-6) and ESAT-6-like protein EsxB, as well as proteins potentially involved in antibiotic resistance acquirement, like putative drug exporters (Tables in the Supplementary material; summarized in Table 3). These findings contribute to a deeper understanding of these microorganisms’ proteomic landscape and biotechnological potential.

**Table 2:** Proteins of potential biotechnological interest identified in the secretome analysis.

Protein	#CBMA	Acession	Coverage (%)	#Peptides	#Unique	Average mass (kDa)
Protease	226	MUL79674.1	6.8	4	4	93.1
Cellulase	226	WP_155898883.1	3.2	3	3	71.0
Amidohydrolase	293	WP_155906880.1	16.1	2	2	29.4
Esterase/lipase	226	WP_155904566.1	30.6	7	5	35.6
	115	WP_155904760.1	36.6	4	4	26.2
	226	WP_208103722.1	22.3	4	4	37.0
	293	MUM13652.1	14.2	3	3	40.1
	294	MUM42711.1	11.4	2	2	35.1
	294	MUM45630.1	11.0	2	2	35.5
Cutinase	293	WP_309224716.1	20.6	3	3	20.3
		MUM14540.1	19.0	3	3	22.0
	294	MUM43196.1	32.9	4	4	22.3
Peroxidase	226	MUL78587.1	39.7	17	17	80.5
	226	WP_155902918.1	43.9	4	4	16.9
Peptidase	293	MUM11921.1	9.9	2	2	37.8
		WP_155900441.1	10.2	2	2	36.9
	293	MUM11762.1	15.6	2	2	28.7

Summarized results from the tables in the Supplementary material, indicating the CBMA isolate where each protein class was detected, the protein accession ID, coverage percentage, number of identified peptides for each sequence (including unique peptides), and the average protein mass. Data were obtained from the database search approach.

**Table 3:** Proteins commonly associated with virulence and pathogenicity in mycobacteria identified in the secretome analysis.

Protein	#CBMA	Peptide	Mass (Da)	NCBI ID
ESAT-related protein	293	SVN(+0.98)SGSSEEQGGSSR	1,467.607	MDZ4267935.1
	294	V(+57.02)SGGTLAAAWGGSGSEAYQGVQTR	2,366.125	MDT5138657.1
Drug exporter	226, 271, 293	VPQLETTSR	1,029.545	MDT5153693.1

Summarized results from Supplementary material indicate the CBMA isolate where each protein class was detected, along with peptide sequences, masses, and protein accession IDs. These data were obtained through a *de novo* sequencing approach.

## 4 Discussion

Identifying and characterizing microorganisms isolated from natural environments has proven paramount across various research disciplines and industrial sectors. These organisms are potential sources of novel enzymatic activities and enzymes with specific catalytic features that may interest diverse industrial and medical applications. Such catalysts can be employed either in their native form or optimized to particular conditions of interest through *in silico* and/or *in vitro* approaches (Ariaeenejad et al., 2024; Ninck et al., 2024). From a public health perspective, natural microbial communities may harbor new pathogens and opportunistic microorganisms, which could have significant implications for the health of human populations. Even in the absence of potentially harmful species, these microbial communities may serve as reservoirs of transferable genetic material for pathogenic bacteria, potentially contributing to the emergence of antimicrobial resistance and virulence, thus constituting part of the local resistome (Fu et al., 2023). Importantly, environmental microorganisms may possess antigens shared with vaccine strains and/or pathogens, as seen in the case of the BCG vaccine and NTM (non-tuberculous mycobacteria) (Brandt et al., 2002; Checkley et al., 2011). The efficacy of the BCG vaccine may vary geographically, being lower in tropical regions, possibly due to increased exposure to NTM (Wilson et al., 1995). Exposure to NTM may impact BCG vaccine efficacy (Flaherty et al., 2006), either by reducing its effectiveness against *M. tuberculosis* infections or by providing partial protection against such infections, depending on factors such as dosage, species, or strain (Young et al., 2007). Therefore, the characterization of microorganisms from CBMA/IOC represents a crucial step in bioprospection for new enzymes and bioactives while contributing to a better understanding of the dynamics of mycobacterial diseases and public health.

The most used culture media for isolation and study of mycobacteria include liquid Middlebrook 7H9 medium and solid media such as Lowenstein Jensen (LJ), Middlebrook 7H10, and Middlebrook 7H11 supplemented with ADC (Noens et al., 2011; Tortoli et al., 2013). However, in this study, LB (Luria-Bertani) medium and LB agar were used for culturing CBMA isolates due to their widespread use in molecular biology procedures, particularly for maintenance and propagation of *Escherichia coli* cultures, and also for culturing other prokaryotes (Miyake et al., 2007). LB medium has also been utilized for mycobacteria procedures, showing similar results to Middlebrook 7H9 for fast-growing mycobacteria like *M. smegmatis* (Stephanou et al., 2007).

Generally, NTMs grow optimally between 25 and 45°C (Tortoli, 2003, 2009; Tortoli et al., 2013). The tested CBMA isolates show minimal growth above 25°C, with optimal growth observed at 22°C. Similar temperature preferences were observed in *M. minnesotense* isolated from a Minnesota swamp, where growth at 28°C was 2 to 3 times higher than at 37°C (Hannigan et al., 2013), and in *M. llatzerense* from a hemodialysis water distribution system in Mallorca, which grew between 22 and 30°C but not at 37 or 45°C (Gomila et al., 2008).

Another significant aspect to investigate in new microorganism specimens is the presence of enzymes that may interest various sectors, due to industrial and medical applications. These catalysts may offer enhanced activity over existing enzymes or possess unique catalytic properties. Mycobrowser (https://mycobrowser.epfl.ch/) states that *M. tuberculosis* H37Rv encodes 28 peptidases, including serine- and metalloendopeptidases. Matrix metalloproteinases (MMPs), a broad family of extracellular enzymes, act on substrates like gelatin (MMP-1 and MMP-9) and collagen (MMP-1, MMP-8, MMP-13, and MMP-18) (Parks and Shapiro, 2000). MMPs have been extensively studied in *M. tuberculosis* due to their increased expression during mycobacterial infections (Sheen et al., 2009; Salgame, 2011; Shiryaev et al., 2011; Rohlwink et al., 2019). NTMs also induce the expression of MMPs, particularly in opportunistic infections (Dezzutti et al., 1999; Swords et al., 2006; Pandey et al., 2012; Roderfeld et al., 2012). All CBMA isolates tested in this study demonstrated proteolytic activity, suggesting the importance of these enzymes during the microorganism’s life cycle.

Another enzyme of interest is cellulase, which is potentially valuable for bioethanol production from agricultural biomass (Ko and Lee, 2018). Cellulose, comprising about half of plant cell walls, is the most abundant carbon source on the planet. While naturally resistant to microbial degradation, certain bacteria such as *Bacillus pumilis*, *Clostridium cellulolyticum*, and *Streptomyces reticuli* produce enzymes capable of hydrolyzing cellulose into glucose (Lynd et al., 2002). Therefore, the discovery of cellulase genes in *M. tuberculosis*, although unexpected given its role in host survival, includes two functional cellulases, Cel6 (or CelA1) and Cel12 (or CelA2), encoded by *rv0062* and *rv1090* genes belonging to GH6 and GH12 families according to the Carbohydrate-Active EnZymes database (www.cazy.org) (Varrot et al., 2005). Additionally, a cellulose-binding protein (CBD2) encoded by *rv1987* and a probable *β*-glucosidase gene encoded by *rv0186* were identified. In *M. tuberculosis*, cellulolytic activity has been shown to play a crucial role in the dynamics of cellulose-based biofilms produced by the bacillus. These biofilms are involved in virulence, antibiotic resistance, colonization capacity, and other features related to the infectious process (Chakraborty et al., 2021). Among 21 mycobacterial genomes analyzed in a study, all NTMs evaluated possessed a Cel6 gene (except *M. ulcerans*), while Cel12 and CBD2 genes varied in presence (zero to five copies) (Mba Medie et al., 2010). It is hypothesized that these enzymes in free-living mycobacteria may be involved in carbon source acquisition, both in environmental niches and symbiotic relationships. This has been previously documented in mycobacterial species interacting with protozoa, where cellulose-rich envelopes are produced during the eukaryotic encystment process (Mon et al., 2024). CBMA isolates were found to express CelA1 homologs, with enzymatic activity observed in CBMA 271 and CBMA 294 isolates by zymography. The presence of cellulase in CBMA isolates isolated from soil suggests its role in their ecological niche. However, further studies are necessary to elucidate the functions of cellulases and peptidases in these microorganisms and explore their potential applications for human benefit.

Another essential aspect to investigate in these isolates is the presence of antigens shared with other mycobacteria, such as the BCG vaccine strain. During the general characterization of these isolates, the search for potential shared antigens (Mpt64, Mpt70, Mpt83, HspX, GlnA1, and Ag85) with the *M. tuberculosis* complex was performed via western blotting with immunodetection. Mpt64 protein was not detected in this analysis, but the gene is present in all four isolates, with duplications in 226, 271, and 293. Mpt64 is a secreted antigen encoded by the RD2 region, present in BCG strains like BCG Moreau, Japan, Russia, and Sweden. It is a candidate for diagnostics in both human and bovine infections (Roche et al., 1996). Mpt70, the major secreted protein of *M. bovis* BCG in Sauton medium (Harboe and Nagai, 1984), stimulates both humoral and cellular immune responses during infections with *M. tuberculosis* or *M. bovis* (Al-Attiyah et al., 2003). Mpt70 was identified in the culture filtrate of all CBMA isolates and the secretomes of 226 and 293. GlnA1 is the principal and essential glutamine synthetase in *M. tuberculosis*, likely playing a role in its survival during infection of the human host (Hayward et al., 2009). This protein, whose encoding gene presents an additional copy in isolate 226, was identified in the total lysates 226, 293, and 294.

The antigen 85 complex consists of three distinct trehalose dimycolate transferases, Ag85A, Ag85B, and Ag85C (encoded by genes *fbpA*, *fbpB*, and *fbpC*), crucial for mycolate deposition and biosynthesis of both mycolic acids and the cell wall, critical for normal mycobacterial cell wall construction and growth (Belisle et al., 1997). Immunoblotting identified the Ag85 antigen in all four environmental mycobacterial isolates; additionally, it was also found in 226, 293, and 294 through secretomics.

It has been already demonstrated that exposure to NTM can compromise BCG vaccine efficacy and influence tuberculosis progression through different mechanisms. One of them is immunological interference, in which individuals with pre-existing high IFN-*γ* responses to PPD (due to NTM exposure) show significantly reduced post-BCG vaccination responses compared to those with lower baseline immunity. This blunted vaccine response suggests NTM exposure may limit BCG’s ability to establish protective immunity (Black et al., 2001; Brandt et al., 2002; Dhanasekaran et al., 2014).

Given that some characteristics of the isolates resemble those of opportunistic/pathogenic species, we decided to investigate intracellular growth kinetics using the THP-1 cell model. Consistent with saprophytic bacterial traits, all isolates exhibited a low internalization rate, but isolate 226 showed significantly different growth kinetics compared to others at longer time points (72 h). These assays are preliminary but suggest the presence of bacteria with varying degrees of virulence in humans in this biome’s soil. Focusing on isolate 226, it was observed to efficiently infect the macrophages under these conditions, indicating the potential for causing harm under specific host conditions.

This study uncovered several indications suggesting these isolates could, in principle, cause opportunistic infections in humans and/or other animals. These include the previously reported genome size of isolate 271 [NCBI ID: VTHB00000000.1 (Morgado and Vicente, 2020)], the ability to proliferate in macrophages observed for isolate 226, and their close phylogenetic proximity to opportunistic NTM species. Our current secretomic analyses also identified proteins involved in virulence and antibiotic resistance acquisition in the *M. tuberculosis* complex. However, further work will be necessary to substantiate these findings.

In the search for biotechnologically interesting enzymes and to expand knowledge about these specimens, several cutinases were identified in the secretome, also confirmed by search in the reported genomes. Cutinases (EC 3.1.1.74) are enzymes originally discovered in phytopathogenic fungi that use cutin as their sole carbon source. Cutin is a complex fatty acid biopolymer that forms the cuticle of higher plants. Cutinases share catalytic properties with lipases and esterases but have the unique ability to be active independently of the presence of a water–oil interface, making them attractive as biocatalysts in various industrial processes involving hydrolysis, esterification, and trans-esterification. Moreover, they exhibit high stability in organic solvents and ionic liquids, enabling their application in industries such as food, cosmetics, fine chemicals, pesticide and insecticide degradation, and treatment of textile industry wastes (Pio and Macedo, 2009; Radley et al., 2023). Cutinases have been used in the degradation of aliphatic and aromatic polyesters (Gamerith et al., 2017), polybutylene succinate (Pan et al., 2018), and others, generating raw material for second-generation ethanol (Duan et al., 2017).

The high number of proteins of unknown function identified in the secretome underscores how much remains to be studied about mycobacteria and their unknown biotechnological potential. The number of hypothetical proteins annotated after a specific search likely results from database updates between genome annotation and this particular search. This amount of hypothetical proteins is not unique to CBMA isolates but is also characteristic of the entire genus (Prasanna and Mehra, 2013).

Characterization of CBMA/IOC microorganisms, focused here on mycobacterial specimens, proves essential for various biological and biomedical research areas. From a public health standpoint, the sharing of antigens between such environmental microorganisms and the BCG vaccine strain may affect the protection conferred by this bioproduct against specific diseases like tuberculosis. On the other hand, such microorganisms can, and often do, serve as sources of new enzymatic activities already used in industry, or they may present some previously unknown metabolic pathway. Thus, continued study of these isolates, as well as others from this and other biological collections from the environment, particularly in Brazil due to its high biodiversity, is highly relevant.

## Data Availability

The original contributions presented in the study are included in the article/Supplementary material, further inquiries can be directed to the corresponding author/s.
