# Supplementary material for: Characterization of mycobacteria isolated from the Brazilian Atlantic Forest: a public health and bioprospection perspective
Source: Front Microbiol. 2025 Apr 25;16:1558006. doi: 10.3389/fmicb.2025.1558006 (PMC12062998; doi:10.3389/fmicb.2025.1558006)
Supplement: Supplementary file 1 [file Data_Sheet_1.docx]

Supplementary Material

## Supplementary Figures


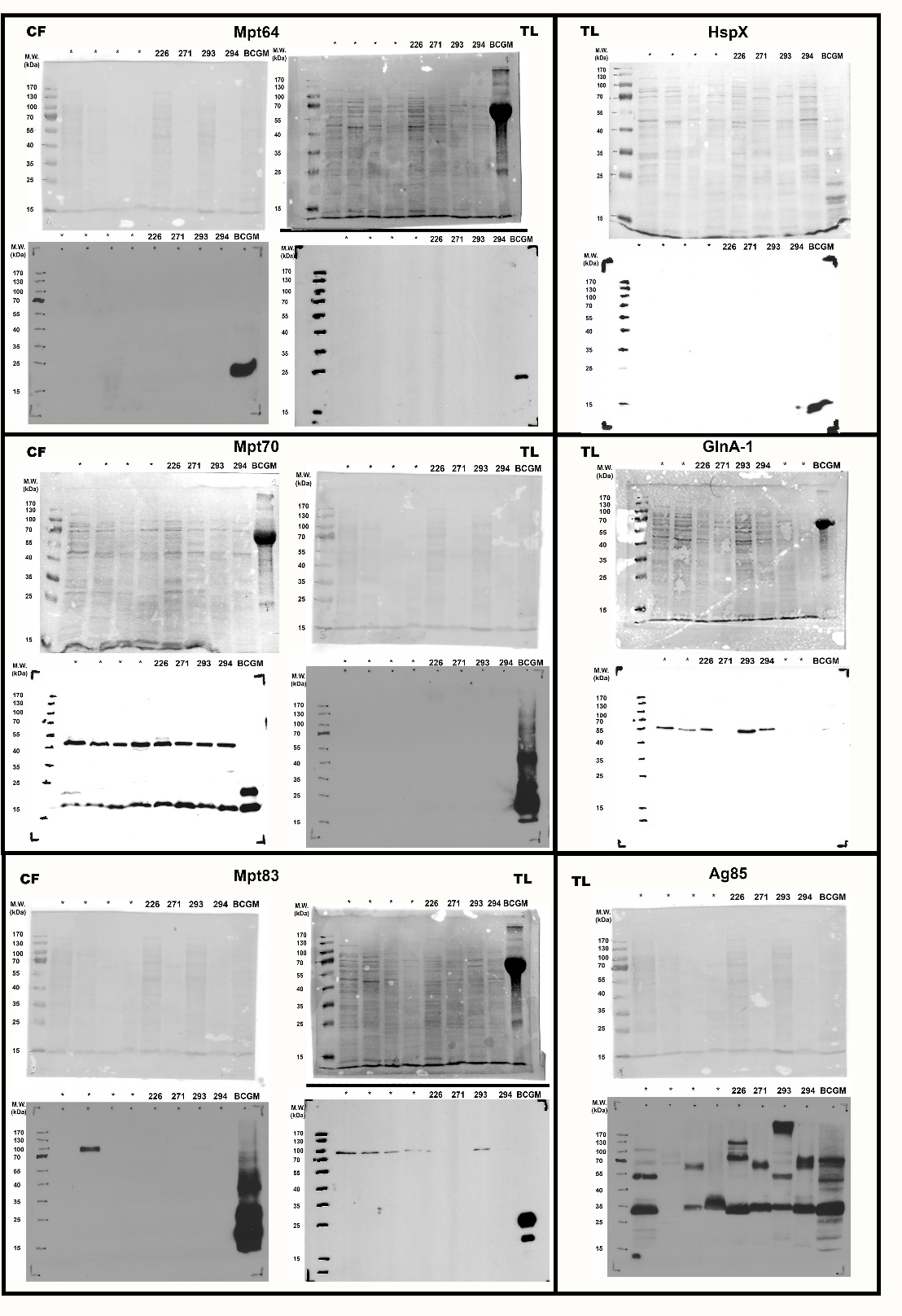


**Supplementary Figure 1.** Detection of Shared Antigens with BCG by western blot. The analysis was performed using total lysate (TL) and/or culture filtrate (CF) protein fractions from all isolates and *M. bovis* BCG Moreau as a positive control. The quality of the electrotransfer and the samples was assessed using reversible MemCode staining (upper panel for each analyzed antigen). Polyclonal sera were previously produced in mice and rabbits against BCG Moreau homologs, including Mpt64, Mpt70, Mpt83, GlnA1, HspX, and Ag85. * Samples not within the scope of this manuscript.

**
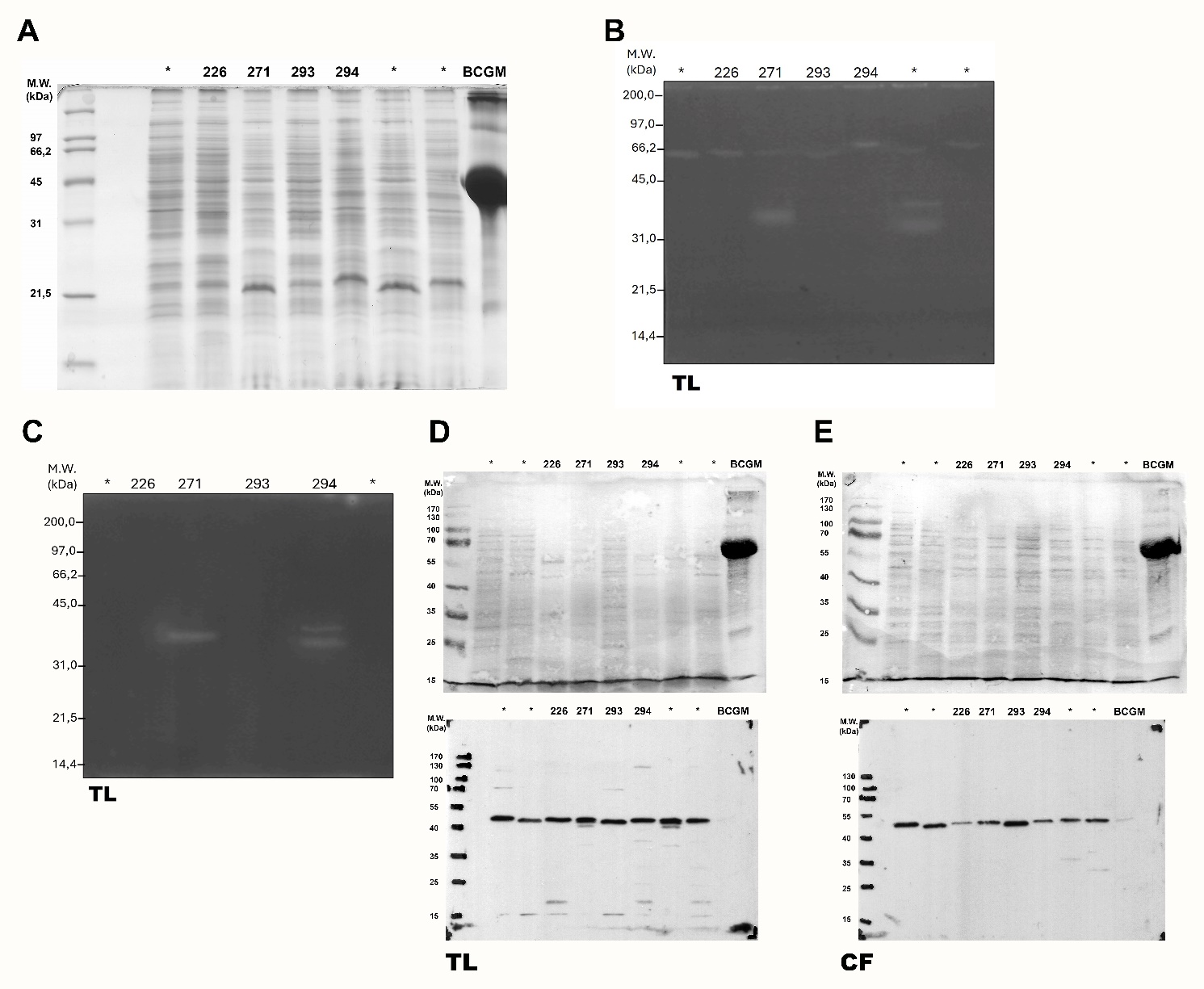
**

**Supplementary Figure 2.** Detection of protease and cellulase in the protein extracts of the isolates. (A) Protein complexity from intracellular fraction of all isolates was analyzed by SDS-PAGE 12% after staining with CBB R-250. Protease (B) and cellulase (C) activity was assessed by zymography with respective substrates in the intracellular protein fraction (total lysate – TL). Cellulase was also detected through western blot assay using α-CelA1 as the primary antibody, both in (D) the intracellular and (E) the culture filtrate (CF) fractions. Prior to western blot, the quality of the electrotransfer and the samples was assessed using reversible MemCode staining (upper panel for each sample). * Samples not within the scope of this manuscript.
